# Editorial: Pathogenesis and Therapy of Graft-versus-Host Disease

**DOI:** 10.3389/fimmu.2019.01797

**Published:** 2019-07-31

**Authors:** Brian C. Betts, Xue-Zhong Yu

**Affiliations:** ^1^Department of Medicine, University of Minnesota Twin Cities, St. Paul, MN, United States; ^2^Department of Microbiology and Immunology, Medical University of South Carolina, Charleston, SC, United States

**Keywords:** All-HCT, GvHD, GvL, T cells, DCs, co-stimulation, metabolism

The therapeutic potential of allogeneic hematopoietic cell transplantation (allo-HCT) for the treatment of malignant diseases relies on the graft-vs.-leukemia (GVL) or graft- vs.-tumor (GVT) responses to eradicate residual tumor cells through immunologic mechanisms. Graft- vs.-host disease (GVHD) is a major cause of transplant-related morbidity and mortality following allo-HCT (allo-HCT). GVHD is clinically described in two forms: acute (aGVHD) and chronic (cGVHD). aGVHD is primarily induced by T cells commonly characterized by a type I T-cell response; whereas cGVHD is induced by both T and B cells, similar in nature to that of autoimmune disorders. Additionally, late acute GVHD, defined as occurring beyond 3 months post-transplant, is associated with high lethality. Despite advances in patient care and pharmacologic prophylaxis strategies, the incidence of GVHD, particularly cGVHD, has not greatly declined over time. In fact, effective treatment options are very limited beyond steroids. Currently, the only FDA-approved agents for steroid-refractory acute and chronic GVHD are ruxolitinib and ibrutinib, respectively. Even with these new agents, GVHD mortality and its impact on quality of life remains a major clinical challenge. Therefore, it is urgently required to further understand GVHD pathogenesis and identify novel therapeutic targets for the prevention and treatment of this devastating disease as a major complication of allo-HCT.

Since allo-reactive donor T cells are central to aGVHD pathophysiology, research has focused on donor T-cell activation, metabolism, co-stimulatory/co-inhibitory signals, differentiation, memory, and migration. Regulation of T-cell allo-responses via protein kinases, metabolites, non-coding RNAs, and other post-transcriptional pathways has also gained substantial attention in recent years. Beyond donor T cells, antigen-presenting cells (APCs) especially dendritic cells (DCs) and other lymphoid cells including natural killer (NK), NKT and innate cells (ILCs) also contribute to aGVHD pathogenesis. In addition, microbiota, tissue injury/repair, and thymopoiesis are also critically involved in aGVHD pathogenesis. The pathophysiology of cGVHD is characterized by fibrosis with inflammation resulting in organ dysfunction. Immunological mechanisms of cGVHD involve (i) aberrant conventional T and B cell activation, differentiation and interactions; and (ii) decreased production and development of regulatory T cells (Tregs). Therefore, cGVHD research is now moving toward a better understanding of the roles of B-cell signaling, activation, germinal center formation, and plasma cell differentiation; as well as the roles of T-cell signaling, activation, and differentiation into T helper and regulatory subsets. In this Research Topic, we brought together six outstanding original research and 10 state-of-the-art Review articles on the pathogenesis and therapy of GVHD that cover the following sub-topics:

## Immune Cells

Significant progress has been made in defining the dichotomous role of DCs in the development of GVHD. Host-derived DCs are important to elicit allogeneic T cell responses, whereas certain donor-types of DCs derived from newly engrafted hematopoietic stem/progenitor cells (HSPCs) can amplify this GVH reaction. In contrast, some DCs also play non-redundant roles in mediating immune tolerance. They induce apoptotic deletion of host-reactive donor T cells while promoting expansion and function of Tregs. Yu et al. focused on the opposing side of the immunologic synapse, and describe how DCs mediate T cell allo-sensitization or immune tolerance after allo-HCT. In an original research paper, the Betts Lab identified a new approach to prevent GVHD that impairs monocyte-derived DC alloactivation of T cells, yet preserves GVL effect Betts et al. They demonstrated that Inhibition of XBP-1 splicing reduces migration of human monocyte-derived DCs, allo-stimulatory potency, and curtails their ability to produce IL-1β, TGFβ, and p40 cytokines, suppressing Th1 and Th17 cell priming without interfering with Treg function or GVL effects by CTL and NK cells.

Original research from the Copsel et al. Lab demonstrates that BET inhibition prevents GVHD, particularly by supporting the expansion of highly potent Tregs. Chen and Mayne describe the limited ability of allospecific CD44^high^ central memory T cells (Tmem) to exert clinical GVHD, owing to functional exhaustion Huang et al. In addition, Shao et al. characterize how ILCs influence GVHD after allo-HCT. Altogether, these primary and review papers delineate the contributions of DCs, Tmems, Tregs, and ILCs in GVHD pathogenesis.

## Cytokine Networks

With regard to inflammatory cytokine networks in GVHD pathophysiology, Piper and Drobyski explore the role of STAT3-dependent inflammatory cytokines in acute GVHD of the gut. They also highlight novel translational approaches to target such cytokines to improve outcomes after allo-HCT. The report from Bastian et al. focuses on the unique anti- and pro-inflammatory properties of the IL-12 cytokine family, and how they impact GVHD onset and severity. Their article features data from pre-clinical studies and early phase clinical trials that identify p40 cytokines and receptor signaling elements, as pathogenic, and thus, candidates for therapeutic intervention. Further, primary research from Yoshihara et al. identifies how TNFα opposes stem cell engraftment. Their work suggests cytokine blockade with etanercept could enhance engraftment and possibly prevent GVHD after allo-HCT. Though a separate clinical entity from GVHD, Senyuk et al. demonstrate that post-transplant hemophagocytic lymphohistiocytosis is similarly driven by alloreactive T cells and inflammatory macrophages.

## Co-stimulation and Co-inhibition

Kumar et al. characterize how co-stimulation or co-inhibitory molecules influence donor T cell allo-activation, including common co-stimulatory molecules such as CD28, ICOS, CD40, CD30, CD27, OX40, and 4-1BB and common negative regulators such as CTLA-4, PD-1, TIM-3, and LAG-3. They discuss how these co-stimulatory and co-inhibitory pathways are involved in T-cell function and contribute substantially in GVHD pathogenesis. They urge that further intensive exploration of these pathways is needed before these potential therapeutic targets could become new clinical options to control GVHD without causing severe side effects. Cassady et al. focus on how PD-L1 interactions with PD-1 and CD80 that differentially regulate auto- and allo-immunity. They discuss how these interactions can separate GVL activity from GVHD in preclinical animal models and highlight the recent clinical application and challenges of PD-L1/PD-1 blockade after HCT for augmenting GVL activity. Original research from the Zhang et al. Lab characterizes the role of ICOS in chronic GVHD pathogenesis as well as its critical influence on Treg biology. They demonstrate that ICOS promotes T- and B-cell activation and differentiation, which can promote cGVHD development; whereas ICOS is also critical for the survival and homeostasis of iTregs, which can suppress cGVHD. Hence, ICOS balances the development of cGVHD and could offer a druggable target to improve clinical outcomes after allo-HCT.

## Metabolism

Metabolism is an attractive therapeutic target to optimize cancer immunotherapy and GVHD prevention. T-cell subsets are poised to distinct metabolic pathways that can determine their function and differentiation. Because distinct T-cell subsets mediate GVH vs. GVL response, the dominant metabolic properties of these distinct subsets might serve as new therapeutic targets that can be exploited to prevent GVHD without compromising GVL activity. Tijaro-Ovalle et al. highlight the metabolic features of malignant hematopoietic cells and discuss the metabolic features that guide the function of T cells and APCs during processes involved in GVH and GVL responses. They also provide rationale for potential therapeutic interventions by targeting metabolic pathways that guide the differentiation and function of these immune cells in the context of allo-HCT. Emerging clinical and pre-clinical evidence indicates that certain micronutrients may participate in regulating GVHD risk after allo-HCT. Dietary micronutrients contribute significantly to modulating various immune responses including cell metabolisms and may influence the susceptibility to autoimmune and inflammatory diseases. Chen and Mayne summarize recent advances in our understanding with respect to the potential role of micronutrients in the pathogenesis of acute and chronic GVHD, focusing on vitamins A and D. They reveal the therapeutic benefits of vitamins A and D in controlling alloreactive T cells.

## The GVL Response

Separating pathogenic GVHD from beneficial GVL is an area of substantial interest and research among the field. In a comprehensive review, Chang et al. summarize the biology of GVH and GVL responses in pre-clinical models and discuss potential novel therapeutic strategies to reduce the relapse rate after allo-HCT. They also review the approaches, including optimal donor selection, conditioning regimens, donor lymphocyte infusion, BCR/ABL-specific CTL, and chimeric antigen receptor-modified T cells, which have been successfully used in the clinic to enhance and preserve the GVL without aggravating GVHD. CTL plays a critical role in mediating the GVL effect. Du and Cao detail how cytotoxic pathways, particularly Fas/Fas ligand, perforin/granzyme, and cytokines, in T cells differentially contribute to GVHD vs. GVL effect.

The collection of articles in “Pathogenesis and Therapy of Graft-vs.-Host Disease” clearly provides an in depth review of our current understanding of GVHD pathophysiology. Moreover, contributions to this collection also present innovative strategies to prevent acute and chronic GVHD, preserve GVL, and support tolerizing Tregs or DCs after allo-HCT.

## Author Contributions

All authors listed have made a substantial, direct and intellectual contribution to the work, and approved it for publication.

### Conflict of Interest Statement

The authors declare that the research was conducted in the absence of any commercial or financial relationships that could be construed as a potential conflict of interest.

